# Bearing Capacity of Reinforced Concrete Beams with Initial Cracks Reinforced with Polymer Composite Materials

**DOI:** 10.3390/polym14163337

**Published:** 2022-08-16

**Authors:** Petr P. Polskoy, Dmitry Mailyan, Alexey N. Beskopylny, Besarion Meskhi

**Affiliations:** 1 Department of Reinforced Concrete Structures, Faculty of Industrial and Civil Engineering, Don State Technical University, Gagarin, 1, 344003 Rostov-on-Don, Russia; 2 Department of Transport Systems, Faculty of Roads and Transport Systems, Don State Technical University, Gagarin, 1, 344003 Rostov-on-Don, Russia; 3 Department of Life Safety and Environmental Protection, Faculty of Life Safety and Environmental Engineering, Don State Technical University, Gagarin, 1, 344003 Rostov-on-Don, Russia

**Keywords:** reinforced concrete, carbon fiber, composite materials, cracks, inclined sections, strength

## Abstract

The use of polymer-composite materials for strengthening the reinforcing of concrete structures represents a current scientific trend. The article is devoted to experimental studies of the strength of inclined sections of bent concrete elements, reinforced with transverse polymer reinforcement with initial inclined cracks, with different shear spans and transverse reinforcement options. The characteristics of reinforced concrete specimens with initial inclined cracks and the test results of 22 experimental beams, each of which was tested twice, are given. A significant influence of all eight variable factors was established: three spans of the section, equal to 1.5 $h_{0}$; 2 $h_{0}$ and 2.5 $h_{0}$; two types of compound clamps and their layout; and opening width of oblique cracks from 0.6 to 0.9 mm. It is shown that the strengthening of the beams supporting sections with external polymer reinforcement using three-sided U-shaped and vertical double-sided stirrups significantly changes their stress-strain state (SSS) and the form of destruction. SSS transforms from the classical destruction of the compressed zone above the end of the inclined crack to the destruction of the beam zone of average height at α = 2.0 and brittle crushing of concrete in the tension zone. Unfavorable combinations of force and geometric factors are revealed. Recommendations are proposed that can be used for structures operated in all weather conditions.

## 1. Introduction

In recent years, the construction industry has been paying more and more attention to the use of polymer-composite materials [1,2,3,4,5,6]. For manufacturing various types of reinforcements, glass and carbon fiber are widely used for internal and external reinforcement of structures. However, applying new engineering decisions and progressive methods should be based on a broad experimental base.

The authors of [7,8,9,10] provide data on the use of internal round glass- and carbon fiber reinforcement as the main reinforcement or in complex reinforced structures with various combinations of steel and composite reinforcement. The work in [11] is devoted to the study of the strength of normal sections of reinforced concrete beams reinforced with external composite reinforcement at various percentages of composite rods. Many studies are associated with the strengthening of compressed elements with external composite reinforcement under various types of stress-strain state. These are conditionally centrally compressed elements, as well as elements operating at small and large eccentricities of the load application [12].

The issues related to the external reinforcement of the supporting sections of the beams under the action of transverse forces are crucial. These studies are partially reflected in the works [13,14]. In 2014, a set of rules for strengthening reinforced concrete structures with composite materials was put in construction field in Russia. This normative document opened the way for the use of these materials as elements of external reinforcement for strengthening structures. However, this document does not provide answers to many questions, including controversial ones. We note that this is also characteristic of the standards of foreign states [15,16,17].

Several issues related to the strengthening of inclined sections of beams on the action of transverse forces are disputable. This concerns the choice of schemes for composite reinforcement of inclined sections for various values of the shear span and the presence or absence of initial cracks.

According to SP 164.1325800.2018, “Reinforcement of Reinforced Concrete Structures with Composite Materials: Design Rules”, the magnitude of the transverse force $Q_{fw}$ in the composite reinforcement is determined by the Formula (1):

(1) $$Q_{fw}=\frac{\psi_{f}\;\left(A_{fw}\times R_{fw}\times \sin\alpha \times C_{fw}\right)}{S_{f}}$$

Here, the coefficient $\psi_{f}$ is used, which considers the type of transverse composite reinforcement. It is 0.95 for closed stirrups and has the same value of 0.85 for U-strap and double-ended stirrup. $A_{fw}$ is cross-sectional area of stirrup made of polymer composite material. $R_{fw}$ is the design value of the tensile strength of the polymer composite material.

It is disputable that the same efficiency of their work is acceptable for several types of stirrups in terms of the degree of anchoring. However, it is known that participation of any reinforcement, longitude or transverse, largely depends on the length and quality of the anchoring zone. In the presence of inclined cracks on the side of the stretched zone, the length of the anchoring zone of the double-sided stirrups near the support is close to zero. In the middle of the cut zone, the anchorage gradually increases. However, near the point of application of the force, it again decreases with the development of an inclined crack in the direction of the vertical load. For U-shaped stirrups, the problem of their anchoring in the tension zone at the beam support is always guaranteed. For double-sided stirrups, there is no anchoring. Therefore, the answer to the question of the reality of the same value of the coefficient $\psi_{f}$ can only be given by experiment.

Building codes completely lack data on the effect of initial inclined cracks and their width on the bearing capacity of inclined sections reinforced with composite materials. However, it is known that all structures during the period of their operation operate at load levels exceeding 0.8 of their limit value, at which both normal and inclined cracks are always formed. The proposals of the standards for injection of cracks are not always fulfilled and do not provide a complete restoration of the continuity of the section.

The use of polymer composite materials to increase the bearing capacity of concrete structures is a relevant subject of research [18,19,20,21,22,23,24,25].

Composite CFRP plates assembled with fiber Bragg grating sensors was developed in [26]. This allowed the authors characterize the dynamic response of the CFRP plate more accurately, and the performance structural characteristics of the two-layer CFRP plates can be tuned using the assembled sensors.

The intelligent composite CFRP-FBG was developed in [27] whose integrity was verified by non-destructive ultrasonic testing. This allowed the authors to identify the interfacial delamination of multilayer structures. The process of degradation of the state of the interfacial bond is associated with variations in the deformation profiles measured by FBG successively in composites.

In the work carried out in [28,29,30,31,32,33,34,35,36,37,38,39,40,41,42], the use of reinforcing bars made of glass-composite polymer, basalt-composite and carbon-composite polymer materials was studied. The efficiency of using polymer composite rebars varies greatly depending on the loading conditions and features of the structure, and experimental studies show the advantages and disadvantages of such a technical solution.

Article [43] addresses the issues of predicting and preventing failures due to separation of carbon composite reinforcement from concrete cover. The authors present a strut model to calculate the critical tensile force leading to failure due to separation of the concrete pavement. Methods for preventing these failures are proposed.

An analysis of studies of the strength of inclined sections of bent reinforced concrete elements, as well as regulatory documents, shows that currently there are no studies carried out according to a single method that would simultaneously concern the strength of inclined sections of beams with initial inclined cracks of various widths that were formed at various shear spans and were again tested after composite reinforcement with various types of stirrups, with a new variation in shear span.

Considering the above, the following tasks were set for this study.

(1) Testing prototypes with initial inclined cracks of various widths, obtained at three shear spans equal to (1.5; 2.0; 2.5) $h_{0}$ and tested after reinforcement with three-sided stirrups at already indicated distances from the support axis to the point of application of concentrated loads.
(2) Obtaining new data on the strength of inclined sections of beams reinforced with external reinforcement under the above variable factors.
(3) Determining the degree of influence of the type of composite stirrups, the scheme of their location, as well as the size of the cut span on the strength of the inclined sections of the prototypes.
(4) Evaluating the effectiveness of composite stirrups at different widths of inclined cracks, both when varying the shear span at the stage of formation of initial cracks, and the final test after strengthening the inclined sections.
(5) Analyzing of the obtained experimental data and evaluate the existing method for calculating beams reinforced with composite materials.
(6) Developing proposals for improving the regulatory framework for the calculation of bending reinforced concrete elements reinforced with composite materials based on carbon fiber under the action of transverse forces.

## 2. Materials and Methods

**Figure 1:**
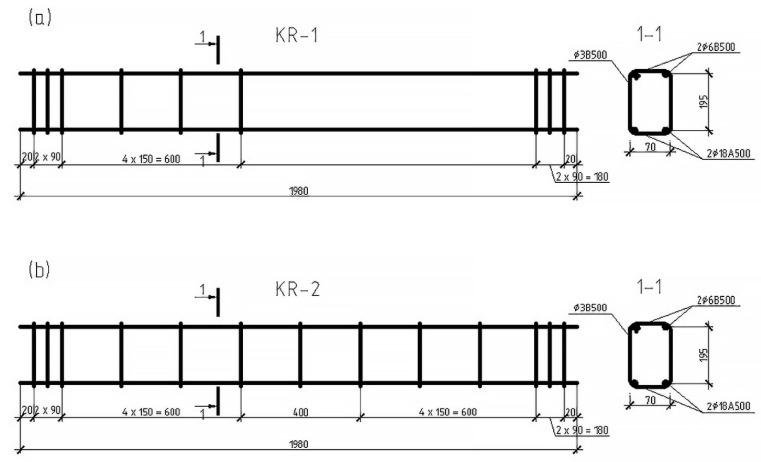
Design for two reference beams: (**a**) ordinary beam and (**b**) reinforced prototypes.

For the experiments, 22 heavy concrete beams with a design class B30, a section of 125 × 250 mm (*h =* 250 mm) and a length of 2.0 m were made. This concrete is now widely used in mass construction. Steel working reinforcement is represented by two rods of a periodic profile Ø18 mm, class A500. The transverse reinforcement consists of stirrups made of cold-formed wire class B500, Ø3 mm. Mounting fittings were adopted Ø6 mm, the similar class. The design of frames for reference and reinforced beams is shown in Figure 1.

It should be noted here that the frame KR-1 and samples Ba-1 and Ba-2 were reference and differed from the rest of the beams by the absence of transverse reinforcement in one of the support sections. This was carried out to determine the magnitude of the transverse force $Q_{b}$ perceived by concrete above the end of an inclined crack and to evaluate the effect of the selected transverse reinforcement on the strength of inclined sections at various shear spans. Based on the results of testing these beams, the expected stages, and levels of loading of ordinary and reinforced prototypes were also determined.

A fabric with a thickness of 0.166 mm and a width of 20 mm based on unidirectional carbon fibers MBraceFibCF 230/4900/300 was used as a composite reinforcement material. The above fabric and related consumables (primer, putty, adhesives) used in surface preparation and gluing the composite were provided by the Moscow branch of BASF Building Systems LLC.

The strength of concrete was determined by the results of testing standard cubes, with an edge of 150 mm (five to seven pieces in each series), according to GOST 10180-90 (2003). Within each series, the spread of cubic strength did not exceed 11.4% and no more than ±7.8% of the average value $\bar{R^{\exp}}$. Concrete class B was determined at standardized values of the reliability index equal to 1.64 and the concrete strength variation coefficient of 0.135. The experimental values of the strength of concrete for axial tension were determined by the cubic strength using the formula laid down in the norms $\bar{R_{bt}}=5\bar{R}/\left(45+\bar{R}\right)$.

**Figure 2:**
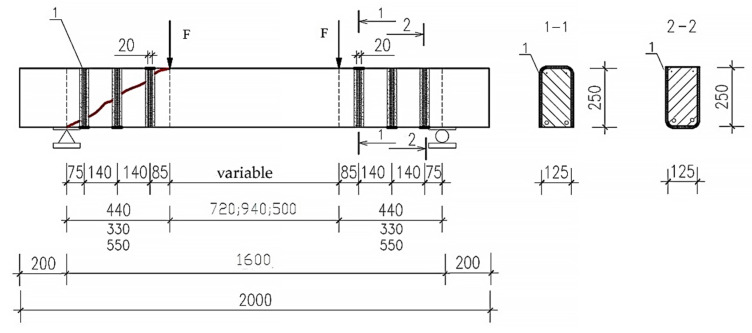
Arrangement of three-way U-shaped composite stirrups for strengthening prototypes.

To reinforce the beams, single-layer three-sided U-shaped stirrups were used, made from the above fabric. The pitch of the composite stirrups was 140 mm. The strength of the stirrups made of carbon fibers according to the results of the tensile test of the figure-of-eight specimens was 3200 MPa. The scheme of composite reinforcement of the supporting sections of the beams is shown in Figure 2.

**Figure 3:**
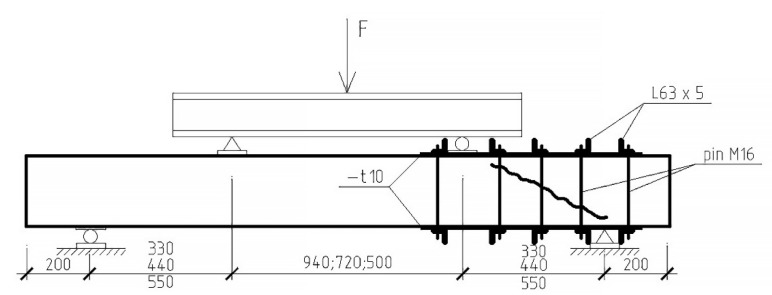
Scheme for testing experimental beams (−t10 means plate with 10 mm thickness).

**Figure 4:**
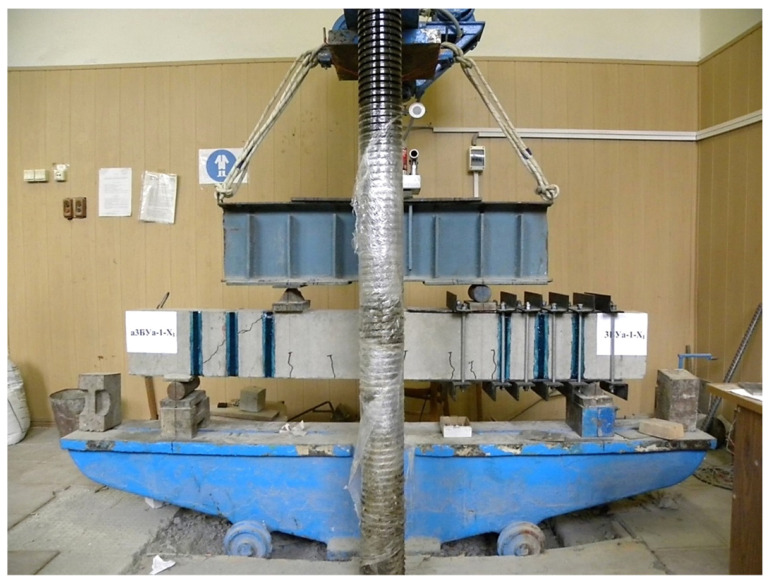
General view of the stand for testing prototypes.

A total of 22 beams were tested, or 44 prototypes, 8 of which were reference (i.e., did not have composite reinforcement). 18 specimens reinforced with U-shaped stirrups out of 36 had initial inclined cracks formed in one of the support sections at three values of the shear span. The second support section was reinforced with a steel hooping during the formation of initial cracks. The scheme of reinforcement with a steel cage and testing of experimental beams is shown in Figure 3. A general view of the stand for testing prototypes is shown in Figure 4.

Samples reinforced with external composite reinforcement, as well as at the stage of cracking, were tested at three different shear spans—1.5 $h_{0}$, 2 $h_{0}$, 2.5 $h_{0}$. The distance from the axis of the supports to the axis of application of the load for each span of the cut is 33, 44, and 55 cm, respectively.

Each beam reinforced with composite stirrups was tested twice with a stepwise increasing load according to the scheme of a single-span hinged beam loaded with two concentrated forces. Prior to testing these beams, one of the support sections was pre-reinforced with a steel cage, according to Figure 3. After the destruction of one of the support sections, without an initial crack, it was also reinforced with a steel cage, after which the beam was re-tested until the destruction of the second end from the initial inclined crack.

This test procedure allows not only doubling the number of samples, but also increasing their reliability, since the method of direct comparison of experimental data allows obtaining reliable results with identical values of concrete strength, sections of steel longitudinal, and transverse reinforcement.

The entire research program was divided into three stages. The first stage involved a preliminary test of 18 samples to obtain initial oblique cracks. The loading was carried out to the level of forces at which the resulting inclined cracks opened up to $a_{arc}$ = 0.6–0.9 mm, which is higher than the maximum allowable crack opening width for normally operated structures and equal to $a_{arc}$ = 0.4 mm. The beams of the first stage are divided into three series—a, b, c (six each in series “b” and “c”, and eight in series “a”). The load in series “a” was applied with a shear span “a” = 2 $h_{0}$, in series “b” = 2.5 $h_{0}$ and series “c” = 1.5 $h_{0}$. Each series provides for the testing of two twin samples. The results of the first stage of the test are shown in Table 1.

At the second stage, before failure, eight reference samples were tested, which do not have external composite reinforcement. These samples, depending on the span of the load shear *a* = (1.5; 2.0 and 2.5) $h_{0}$, are also divided into three series. These tests provide data on the magnitude of the shear force taken by the concrete in the compressed zone above the end of the inclined crack ($Q_{b}$) and the shear force perceived by the steel transverse reinforcement crossed by the inclined crack ($Q_{sw}$).

At the third stage, it was planned to test beams reinforced along inclined sections with U-shaped vertical stirrups made of carbon fabric, with a width of $W_{f}$ = 20 mm, located with a step in the axes of 140 mm. The clear distance is 120 mm, which does not exceed the recommended values: $h_{0}$/2 = 125.

It should be noted that the first two stirrups at the support were located from bottom to top. The third clamp from the support, located next to the force, was glued from top to bottom. This prevents shearing of the concrete above the end of the inclined crack at the moment of sample failure.

At this stage, the effectiveness of the composite reinforcement was clarified in comparison with the reference samples (at different values of the shear span).

From what has been said, it follows that the prototypes having a crack formed, for example, at a shear span of 2.0 $h_{0}$, after strengthening, were tested at three different shear spans *a* = (1.5; 2.0 and 2.5) $h_{0}$. The external transverse reinforcement remained the same. Similarly, prototypes were tested, in which initial cracks were obtained at shear spans equal to 1.5 $h_{0}$ and 2.5 $h_{0}$. The results of the second and third stages of testing are shown in Table 2.

In a separate (fourth) stage, eight prototypes were tested, reinforced with double-sided stirrups, which have a steel frame structure similar to the considered beams, as well as the cross section and pitch of composite stirrups. They were not included in this article. However, they were used when comparing the results of the effectiveness of various amplification options.

## 3. Results and Discussion

Starting to evaluate and analyze the results of testing prototypes, it should be noted that during the experiments, eight parameters were varied, namely three values of the shear span, two types of composite stirrups and their layout, as well as the presence or absence of initial or inclined cracks, the width of which ranged from 0.6 to 0.9 mm. Direct comparison of the experimental results, considering the concrete strength reduced to the reference samples, shows that each of the variable factors has a significant effect on the bearing capacity of inclined sections of reinforced concrete beams reinforced with composite materials.

Considering that the title of the article focuses on the initial inclined cracks, the effect of the stirrups type and the shear span on the bearing capacity of reinforced sections is presented mainly in the form of conclusions. These data were used in a comparative assessment of the effectiveness of composite stirrups obtained from the results of the experiment.

Further analysis showed that due to the spread of concrete strength (see column 5 of Table 2) there was no clear picture of the influence of each of the above variable factors. So, further analysis was performed using the given strength characteristics of prototypes to the strength of concrete reference beams.

The transverse force perceived by the steel transverse reinforcement did not change in this case (see column 11 of Table 2).

Let us sequentially consider the influence of each of the listed factors.

**Figure 5:**
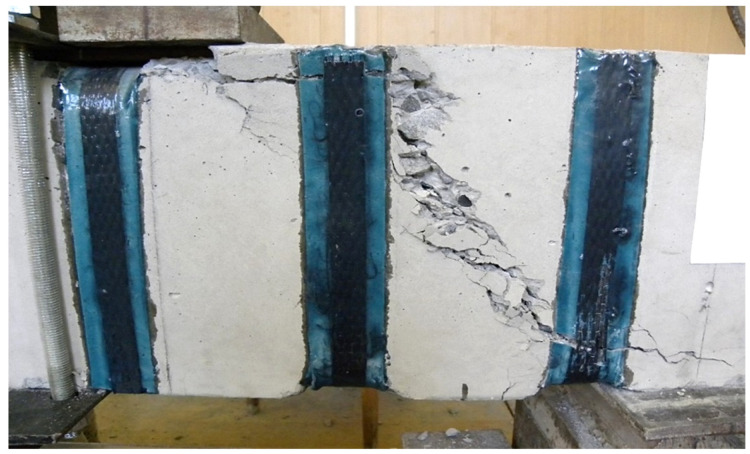
The nature of the collapse of the prototype No. 29, reinforced with U-shaped stirrups during the cut 1.5 $h_{0}$.

**Figure 6:**
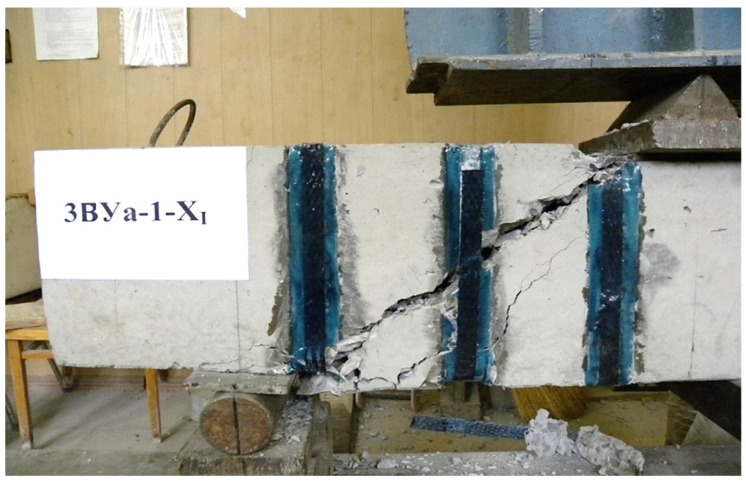
The nature of the collapse of the prototype No. 34, reinforced with U-shaped stirrups during the cut 2.0 $h_{0}$.

**Figure 7:**
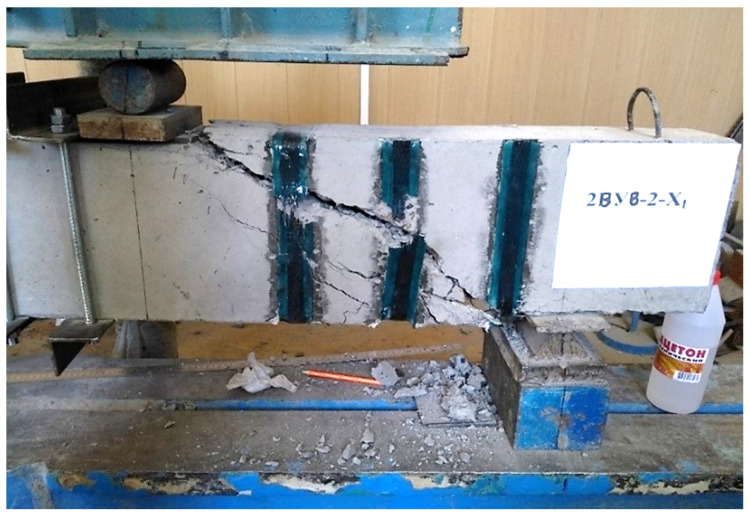
The nature of the collapse of the prototype No. 40, reinforced with U-shaped stirrups during the cut 2.5 $h_{0}$.

It should be noted that all prototypes, both reference and reinforced with carbon fabric, collapsed along an inclined section. There were only the following differences related to the length of the shear span:

(1) Inclined cracks in the reinforced samples appeared at higher load levels compared to the reference samples. Moreover, with U-shaped stirrups, it was slightly higher. With double-sided stirrups, the load level during the appearance of cracks was somewhat lower.

(2) The trajectory of the development of inclined cracks with a shear span of *a* =1.5 $h_{0}$ gravitated towards the support reaction. At *a* = 2.0 $h_{0}$–was within the distance of the support plates at the support and under force; at a shear span of 2.5 $h_{0}$, the crack moved in the direction of the action of the concentrated force, sometimes entering the zone of pure bending. The nature of the destruction of prototypes reinforced with U-shaped stirrups at shear spans 1.5 $h_{0}$, 2 $h_{0}$ and 2.5 $h_{0}$ is shown in Figure 5, Figure 6 and Figure 7.

(3) The projection of cracks on the horizontal axis in the reinforced samples at each span of the cut was less than in the reference ones. In this case, the length of this projection in the reference and reinforced samples tends to increase with an increase in the span of the cut. However, it never exceeded the value of 2.0 $h_{0}$.

(4) The reference samples were destroyed smoothly, from cutting the concrete of the compressed zone or crushing it above the end of an inclined crack. The specimens reinforced with U-shaped stirrups fractured brittlely and volumetrically almost along the entire length of the crack. Their feature is the destruction of the inclined section from the crushing of concrete in the stretched zone, which is shifted towards the action of the force with an increase in the span of the cut. For double-sided stirrups, this feature is absent.

(5) Three-sided U-shaped stirrups, glued from bottom to top at the support and from top to bottom near the application of the load, have a more significant effect on the bearing capacity of inclined sections compared to vertical stirrups, the bearing capacity of which is almost two times lower.

(6) The presence of initial cracks with an opening of 0.6–0.9 mm leads to a sharp decrease in the bearing capacity and efficiency of U-shaped stirrups $Q_{f}$, which simultaneously decreases with a decrease in the shear span. For double-sided stirrups, the transverse force $Q_{f}^{*}$ tends to zero.

The general view and nature of the destruction of prototypes with initial inclined cracks, formed and tested at three values of shear spans, are shown in photos No. 5–7.

(7) The effectiveness of external composite reinforcement of inclined sections largely depends on the span of the cut, during which inclined cracks were formed. The smallest value of $Q_{f}^{*}$ was obtained when the shear span equal to 1.5 $h_{0}$ coincided both during the formation of the initial crack and during testing of the reinforced specimen. When the span of the cut coincided *a* = 2.5 $h_{0}$, the influence of the initial cracks was less significant.

The results presented in Table 3 cannot be considered without evaluating the operation of the U-shaped and double-sided stirrups glued to the side surfaces of the prototypes. An estimate of the effectiveness of this amplification can be formulated as follows.

The bearing capacity of transverse composite stirrups decreases with decreasing shear span. This is explained by the fact that, firstly, with a small shear span, inclined cracks cross a smaller number of composite stirrups. Secondly, with an increase in the angle of inclination of cracks, the tensile strength of concrete is gradually transferred to the work of concrete in shear, at which it is greater than pure tension.

A direct comparison of the transverse force perceived by U-shaped stirrups in samples without initial inclined cracks, on average, regardless of the shear span, is 31–37% lower than in their absence. The effectiveness of double-sided stirrups in samples with initial cracks is lower by 80% compared to reinforced samples without cracks. This is due to the fact that none of the double-sided stirrups broke during the tests, since destruction along the inclined section occurred due to the separation of the stirrups from the beam along the concrete body. This is mainly due to two factors: the width of the opening of the initial inclined cracks and the absence of the required length of the anchoring zone for double-sided composite stirrups, which is equal to the distance from the crack face to the end of the composite clamp in the direction of the compressed or tensioned zone of the beam. This is especially important for beams with a small height, when the length of the anchoring, glued clamp on one side or the other of the crack, will almost always be lower than the minimum value of the length of this zone, equal to 150 mm. However, most standards do not consider this factor.

The magnitude of the transverse force $Q_{fw}$ perceived by the composite reinforcement in Russian standards is determined by Formula (1), in which the influence of the type of external stirrups is estimated using the coefficient $\psi_{f}$. For closed stirrups, this coefficient is equal to 0.95, and for three-sided U-shaped and vertical two-sided stirrups, it is assumed to be the same and equal to $\psi_{f}$ = 0.85.

In our experiments, as noted above, U-shaped stirrups were glued from bottom to top in the support areas and from top to bottom in the zone of application of loads. This allows you to dramatically increase the rigidity of the upper zone of the beams, since, without breaking the stirrups, the concrete above the end of the inclined crack cannot be sheared. The clamp cannot come off the side surface, since before the destruction of the sample, inclined cracks are located at the top of the beam and have a sufficient anchoring length below the crack, more than 15 cm.

Experiments confirm that the effectiveness of a U-shaped stirrup, glued next to a concentrated load from top to bottom, is located an intermediate position between a closed stirrup and a U-shaped stirrup, glued from bottom to top. This gives grounds to accept the value of the coefficient $\psi_{f}$ as an average value between 0.95 for a closed and 0.85 for a three-sided hoop (i.e., takes the value $\psi_{f}$ = 0.9). This is confirmed by the results of a comparison of the transverse forces perceived by U-shaped and vertical double-sided stirrups in beams with and without initial cracks, at different shear spans. The ratio $\bar{Q_{f}}/\bar{Q_{f}^{*}}$ for three-sided stirrups is on average 0.64, and for two-sided 0.19.

These data support the fact that initial oblique cracks drastically reduce the effectiveness of composite reinforcement, especially when double-sided stirrups are used. The latter confirms the need to correct the methodology of the standards.

It should also be noted that the experimental value of $Q_{fw}$ for U-shaped collars is almost twice its calculated value and equal to $Q_{fw}^{theor}$ = 16.75 kN for elements tested at shear span, a = 2.0 $h_{0}$ and 2.5 $h_{0}$. With a = 1.5 $h_{0}$, $Q_{fw}^{theor}$ = 12.6 kN. These figures are in good agreement with the average value $\bar{Q_{f}}$ = 17.6 kN for cracked reinforced beams.

### 3.1. Influence of Shear Span and Type of Composite Stirrups

Based on the results of Table 2, the values of three quantities that make up the bearing capacity of the reinforced samples $Q_{f}$ were determined. This quantity consists of the sum of three terms $Q_{b}+Q_{sw}+Q_{fw}$. In fact, the bearing capacity of a reinforced section can be represented as two terms, consisting of the non-existent capacity of a reinforced concrete beam and the bearing capacity of external stirrups for U-shaped ones and for vertical double-sided ones.

The intensity of the transverse reinforcement in the prototypes $q_{sw}$, was the same and amounted to $q_{sw}$ = 3 76 N/cm, and the most unfavorable value of the projection of the inclined section “$C_{0}$” on the horizontal axis is 66.45–75.0 cm. This is much more than the maximum allowable value $C_{0}$ = 2.0 $h_{0}$ = 44 cm, regulated by the norms. Therefore, the theoretical strength of inclined sections in the margin of safety was determined at $C_{0}$ = 33 cm for elements tested with a shear span a = 1.5 $h_{0}$ and $C_{0}$ = 44 cm for elements with a shear span of 2.0 $h_{0}$ and 2.5 $h_{0}$.

The adopted analysis parameters allow, with the same reliability for all prototypes, to determine the experimental-theoretical strength perceived by external composite transverse stirrups

(2) $$Q_{fw,theor}^{\exp}=Q_{ult}^{\exp}-{\left(Q_{b}+Q_{sw}\right)}^{theor}$$

This assumption allows, with a high degree of reliability, using Formula (1), to determine the transverse force perceived by transverse reinforcement made of composite materials in an inclined section, to estimate the theoretical value, and, if necessary, to make suggestions to refine the calculation method.

The experimental values of the transverse forces perceived by the U-shaped collars are shown in Table 3.

### 3.2. Accounting for the Influence of Opening Width of Initial Oblique Cracks

To quantify the effect of the opening width of the initial inclined cracks on the $Q_{f}^{theor}$ value perceived by the composite reinforcement, in Figure 4, using the symbols shown in Table 3, the dependence of the $Q_{f}$ value on the opening width of the initial inclined cracks and the average value of $Q_{f}$ and $Q_{f}^{*}$ at different shear spans.

The horizontal section of this graph corresponds to the allowable width of inclined cracks $a_{arc}$ = 0.3 mm, and the inclined lines correspond to the change in the value of $Q_{f}$ for different shear spans with a change in the width of the initial inclined cracks. The figure shows a clear effect of the width of the opening of inclined cracks at different spans of the cut.

**Figure 8:**
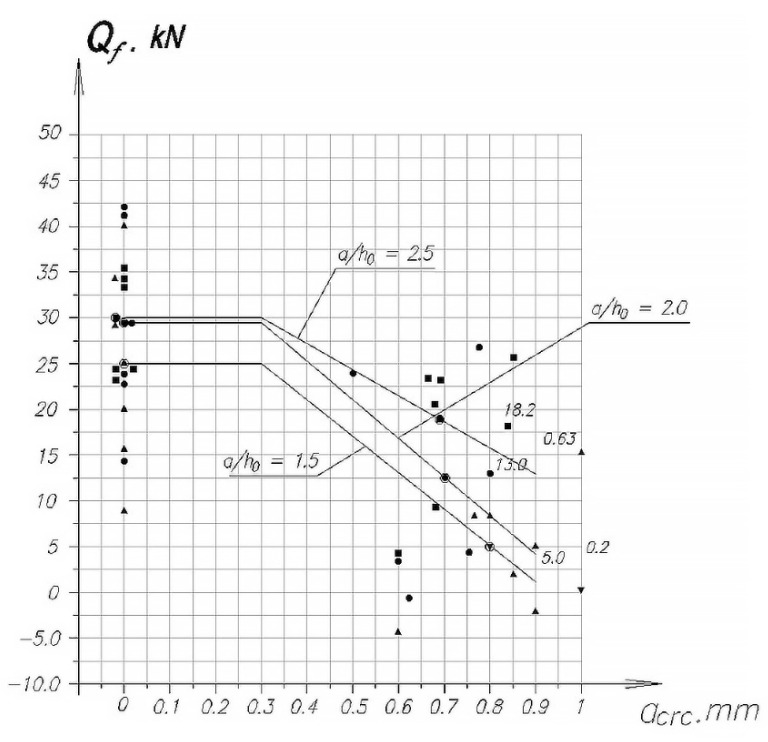
Influence of the opening width of the initial oblique cracks $a_{arc}$ on the effectiveness of composite reinforcement at different shear spans (symbols see Table 3).

**Figure 9:**
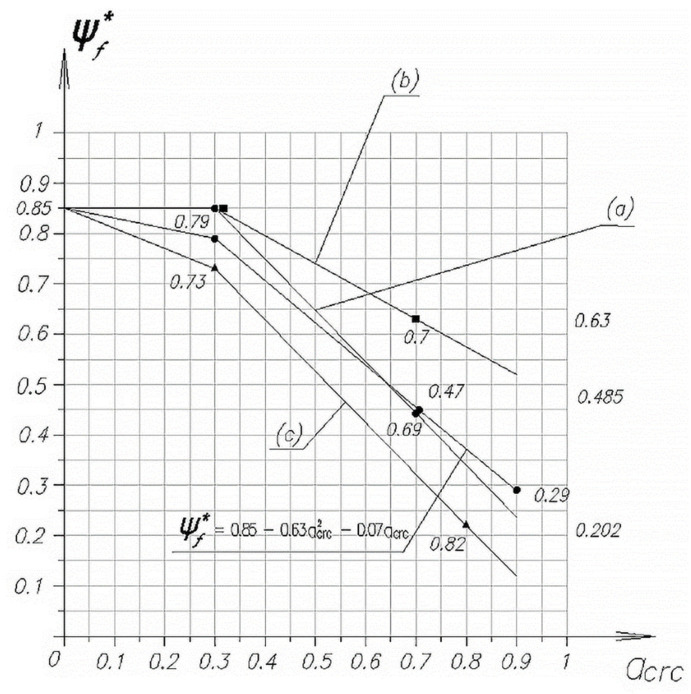
Change in the coefficient $\psi_{f}^{*}$ for three-sided U-shaped stirrups depending on the width of the opening of inclined cracks formed during the passage of the cut: (**a**) *a* = 1.5 $h_{0}$; (**b**) 2.0 $h_{0}$; (**c**) 2.5 $h_{0}$.

To obtain specific data on the effect of the width of the opening of inclined cracks on the value of $Q_{f}$, in Figure 8 and Figure 9 for the purpose of using in the calculations, graphs of the change in the relative value of $Q_{f}^{*}$/$Q_{f}$ are presented, depending on the width of the opening of the initial cracks in the form of dependence $\psi_{f}^{*}=F(a_{arc})$.

The average value of $\psi_{f}^{*}$, regardless of the span of the cut, is approximated well by the analytical expression of this coefficient.

(3) $$\psi_{f}*=0.85-0.63\times a_{crc}^{2}-0.07\times a_{crc}$$

Based on the results of the analysis performed, the following proposals can be made to improve the calculation technique:

(1) The assessment of the bearing capacity of concrete in an inclined section should be carried out using the coefficient $\varphi_{b2}$ = 2.0 instead of $\varphi_{b2}$ = 1.5 adopted in the Code of Rules, at which the bearing capacity of composite stirrups is artificially overestimated, which further leads to a decrease in the bearing capacity of the reinforced sections.

(2) It is necessary considering the effect of the shear span on the bearing capacity of the inclined section by introducing into the formula for determining the value of $Q_{b}$, the coefficient $k_{\varphi_{b2}}$, the value of which is recommended to be determined by the proposed formula:

(4) $$k_{\varphi_{b2}}=\sqrt{2\;h_{0}/a}$$

(3) Three-sided U-shaped collars and vertical two-sided should be evaluated by the same coefficient $\psi_{f}$ = 0.85. However, in our case, even with a minimum percentage of composite reinforcement, they have a different degree of reliability. Therefore, the formula for determining the value of $Q_{f}$, the value of the coefficient $\psi_{f}$ should be differentiated, depending on their type and layout in an inclined section, as well as depending on the height of the bent elements and the presence or absence of inclined cracks.

(4) In the absence of initial inclined cracks, the arrangement of U-shaped stirrups, when they are glued from the bottom up at the support, and from top to bottom in the zone of application of concentrated forces, due to which they sharply increase the rigidity of the inclined section, this factor must be taken into account additional increasing coefficient $\psi_{f}$ = 0.9, which occupies an intermediate position between the clip and three-sided stirrups, glued only from the bottom up.

(5) In the presence of initial inclined cracks, instead of a fixed value of the coefficient $\psi_{f}$ = 0.85, a differentiated coefficient $\psi_{f}^{*}$ should be introduced, which varies with the length of the shear span, and, on average, is well correlated with the following dependence (3).

(6) Coefficient $\psi_{f}^{*}$ can be represented as a product of two coefficients, regulated $\psi_{f}$ and differentiated $k_{\psi_{f}}$. The value of the coefficient $k_{\psi_{f}}$, depending on the opening width of the initial inclined cracks and the size of the shear span, can be determined by the formulas:

(5) $$a=1.5\;h_{0}:\;k_{\psi_{f}}=1.19-1.14a_{crc}$$

(6) $$a=2.0\;h_{0}:\;k_{\psi_{f}}=1.36-1.2a_{crc}$$

(7) $$a=2.5\;h_{0}:\;k_{\psi_{f}}=1.2-0.65a_{crc}$$

(7) Consider the decrease in the efficiency of vertical double-sided stirrups for elements up to 450 mm high by the factor $\psi_{f}$ = 0.6.

(8) In the presence of initial inclined cracks with an opening of more than 0.4 mm, the use of double-sided stirrups is not recommended.

(8) $$k_{\psi_{f}}=1.2-0.65a_{arc}$$

(9) In the presence of an initial inclined crack with an opening of 0.5 mm, formed during the span of the shear a = 1.5 $h_{0}$, the reinforcement of the support section should be performed using a solid or U-shaped two-layer clip within the horizontal projection of the inclined crack with an additional anchoring zone of 150 mm in beginning and end of an inclined crack.

(10) Considering that at shear spans equal to 2.5 $h_{0}$ during testing, inclined cracks go beyond the point of application of a concentrated load, for structural reasons it is proposed to install another step of composite stirrups with an arrangement from top to bottom, located already in the zone of pure bending.

Considering all of the above, the final formulas for calculating the strength of inclined sections in the presence and absence of initial inclined cracks will take the following form:

(9) $$Q=Q_{b}+Q_{sw}+Q_{fw}$$

(10) $$Q_{b}=\frac{k_{\varphi_{b2}}\times \varphi_{b2}\times R_{bt}\times h_{0}^{2}}{C}$$

In the absence of initial cracks:

(11) $$Q_{fw}=\psi_{f,1-4}\frac{A_{fw}\times R_{fw}\times C_{fw}}{S_{f}}$$

With initial cracks:

(12) $$Q_{fw}^{*}=\psi_{f}^{*}\frac{A_{fw}\times R_{fw}\times C_{fw}}{S_{f}}$$

Regardless of the presence or absence of initial cracks:

(13) $$Q_{fw}=\psi_{f}\times k_{\psi_{f}}\times \frac{A_{fw}\times R_{fw}\times C_{fw}}{S_{f}}$$

For the final verification of the adopted recommendations, a verification calculation of the bearing capacity of inclined sections was performed according to the recommendations of the Russian standards, considering the authors’ proposals. Comparison of experimental and theoretical values of the strength of inclined sections reinforced with composite materials is presented in Table 4.

## 4. Conclusions

(1) As a result of comprehensive studies of concrete beams reinforced with external composite reinforcements, new experimental data were obtained on the strength of inclined sections of bending elements in the presence and absence of initial inclined cracks, with different shear stirrups and transverse reinforcement options.

(2) Reinforcement of the bearing sections of the beams with external transverse reinforcement using three-sided U-shaped and vertical double-sided stirrups significantly changes their stress-strain state and the form of destruction. The latter passes from the classical destruction of the compressed zone above the end of the inclined crack with a shear span of 2.5 $h_{0}$ to the destruction of the middle-height zone of beams with α = 2.0 and brittle crushing of concrete in the tension zone with a shear span of 1.5 $h_{0}$.

(3) The presence of initial inclined cracks, especially with an opening of more than 0.6 mm, reduces the bearing capacity with a decrease in the shear span taken during the test. The most unfavorable combination is when the acting load is applied both in the process of initial crack formation and in testing reinforced samples, with the same shear span equal to 1.5 $h_{0}$. The magnitude of the transverse force $Q_{f}$ perceived by the composite reinforcement directly depends on the width of the opening of inclined cracks, and the greater, the smaller the shear span.

(4) The coefficient $\psi_{f}$, which considers the type of external composite clamps, which is a fixed value and equal to 0.95 or 0.85 for reinforced elements without initial cracks, turns into a differentiated one for elements with initial inclined cracks. Its value depends both on the width of the opening of inclined cracks and on the size of the shear span.

The recommendations proposed as a result of the study can be used for structures operated in all weather conditions. This article does not exhaust the entire range of issues related to improving the methodology for calculating inclined sections of reinforced concrete beams reinforced with composite materials. Research will be continued with the aim of further studying issues that relate to the support sections of beams with shear spans of 1.5 $h_{0}$ or less.

## Figures and Tables

**Table 1:** Characteristics of prototypes with initial inclined cracks formed at different shear spans.

Test Stage	Shear Span during Initial Crack Formation a, cm	Code of Beams during Initial Crack Formation	Ordinal Number of Specimens after Reinforcement	$\mathrm{Cubic}\;\mathrm{Strength}\;\mathrm{of}\;\mathrm{Concrete}\;\bar{R},\;\mathrm{MPa}$	Shear Force at:, kN		$\mathrm{Width}\;\mathrm{Cracks}\;a_{arc}\;\mathrm{at}:,\;\mathrm{mm}$			Horizontal Projection Incl. Cracks “C”, cm
					Crack Formation Q_cr_	Max. Crack Opening Q	Formation	Max. Crack Opening	After Unloading.	
1	2	3	4	5	6	7	8	9	10	11
Samples reinforced with three-way U-stirrups										
I stage	$2\;h_{0}$	a1B	14		25.0	37.5	0.02	0.68/0.60	0.48/0.40	32.0
		a2B	16	301.3	20.0	32.5	0.08	0.70/0.60	0.48/0.40	25.0
		a3B	10		30.0	52.5	0.02	0.64/0.70	0.40/0.48	31.0
		a4Б	12	362.3	30.0	45.0	0.08	0.75/0.7	0.45/0.30	29.0
		a5B	18		25.0	35.0	0.10	0.80/0.63	0.48/0.30	30.0
		a6B	20	259.4	20.0	32.5	0.07	0.85/0.50	0.45/0.20	29.0
	$2.5\;h_{0}$	b1B	30		30.0	40.0	0.08	0.60/0.40	0.30/0.20	25.0
		b2B	32	342.5	25.0	35.0	0.06	0.75/0.30	0.50/0.20	27.0
		b3B	22		35.0	49.0	0.06	0.60/0.75	0.40/0.45	28.0
		b4B	24	373.8	35.0	40.0	0.085	0.80/0.70	0.45/0.55	24.0
		b5B	26		25.0	45.0	0.07	0.85/0.60	0.65/0.40	21.0
		b6B	28	391.5	35.0	60.0	0.06	0.66/0.60	0.40/0.45	25.5
	$1.5\;h_{0}$	c2B	38		35.0	62.5	0.10	0.60/0.70	0.25/0.38	13.5
		c1B	40	383.0	37.5	85.0	0.08	0.70/0.54	0.35/0.20	16.0
		c3B	34		35.0	67.5	0.08	0.64/0.56	0.40/0.30	12.5
		c4B	36	335.2	35.0	77.5	0.09	0.70/0.40	0.30/0.12	15.0
		c5B	42		40.0	80.0	0.06	0.90/0.40	0.50/0.10	13.0
		c6B	44	311.8	35.0	70.0	0.04	1.0/0.50	0.50/0.40	12.0

Notes: (1) The denominator of columns 9 and 10 shows the width of the opening of inclined cracks for the reverse side of the beams. (2) The age of the samples from the moment of manufacture to the test was 4.5 years ± 2 months.

**Table 2:** Results of the experiment on the efficiency of composite reinforcement of inclined sections.

Shear Span at:		N of Samples	Experimental Values:					$\mathrm{Average}{\bar{Q}}_{b},{\bar{Q}}_{sw,b},{\bar{Q}}_{f}\mathrm{kN}$
Forming Inclined Cracks	Testing Etalon or Enhanced Samples		Concrete Strength	Shear Force				
			Compressive, MPa/Tensile R_bt_, MPa	$\mathrm{At}\;\mathrm{Collapse}\;Q_{ult}^{exp};Q_{ult}^{exp*},\;\mathrm{kN}$	$\mathrm{In}\;\mathrm{Compressed}\;\mathrm{Concrete}\;Q_{fb}$ $,\;\mathrm{kN}/\mathrm{Transversal}\;\mathrm{Reinforcement}\;Q_{sw},\;\mathrm{kN}$	$\mathrm{Reduced}\;\mathrm{Concrete}\;\mathrm{Strength}\;Q_{fb}^{red}$ $,\;\mathrm{kN}/\mathrm{Strength}\;\mathrm{of}\;\mathrm{the}\;\mathrm{Inclined}\;\mathrm{Sec}\mathrm{tion},\;\mathrm{Considering}\;\mathrm{the}\;\mathrm{Reinforcement}Q_{ult}^{red},\;\mathrm{kN}$	In Polymer reinforcement Q*_f_*, kN	
1	2	3	4	5	6	7	8	9
Etalon samples								${\bar{Q}}_{b};{\bar{Q}}_{sw,b}$
-	$2\;h_{0}$ (series a)	1	28.5/2.24	74.1	-/12.59	-/-	-/-	79.55
		2		85.0		-/-	-/-	
		3		90.0		-/-	-/-	92.125
		4		94.25		-/-	-/-	
-	$2.5\;h_{0}$(series b)	5	28.0/2.22	81.3	-/13.35	-/-	-/-	77.9
		6		74.5		-/-	-/-	
-	$1.5\;h_{0}$(series c)	7	27.0/2.18	112.0	-/12.22	-/-	-/-	102.75
		8		93.5		-/-	-/-	
**Samples reinforced with U-stirrups**								${\bar{Q}}_{f}$
2*h*_0_ (a)	$2\;h_{0}$ (series a)	9	28.2/2.23	113.6	101.01/12.59	101.46/114.05	21.93	32.4
		10		95.0	82.41/12.59	82.78/95.4	3.28	
		11		134.5	121.91/12.59	122.46/135.5	42.93	
2*h*_0_ (a)		12	28.2/2.23	120.0	107.41/12.59	107.89/120.48	28.36	15.8
	$2.5\;h_{0}$ (series b)	13	23.5/2.01	101.3	87.95/13.35	97.14/110.49	32.59	28.15
		14		93.5	80.15/13.35	88.52/101.87	23.97	
		15		93.25	79.9/13.35	88.25/101.6	23.7	
		16		90.0	76.63/13.35	84.65/98.0	20.1	22.0
	$1.5\;h_{0}$ (series c)	17	20.2/1.83	105.0	92.78/12.22	110.5/122.7	19.99	18.2
		18		95.5	83.28/12.22	99.2/111.4	8.65	
		19		102.0	89.78/12.22	106.95/119.2	16.45	
		20		90.0	77.78/12.22	92.65/104.9	2.15	5.4
2.5*h*_0_ (b)	$2\;h_{0}$ (series a)	21	29.9/2.27	115.0	102.41/12.59	101.05/113.65	21.52	21.0
		22		115.0	102.41/12.59	101.05/113.65	21.52	
		23		114.0	101.41/12.59	100.06/112.66	20.53	
		24		108.0	95.41/12.59	94.15/106.74	14.6	18.1
	$2.5\;h_{0}$ (series b)	25	26.1/2.13	109.45	96.1/13.35	100.16/113.5	35.6	34.3
		26		102.0	88.65/13.35	92.4/105.75	27.85	
		27		107.0	93.65/13.35	97.6/110.95	33.05	
		28		84.0	70.65/13.35	73.64/87.0	9.1	18.5
	$1.5\;h_{0}$ (series c)	29	26.7/2.16	110.0	97.78/12.22	98.7/110.9	8.15	18.5
		30		96.0	83.78/12.22	84.56/96.77	−6.0	
		31		130.5	118.28/12.22	119.4/131.6	28.85	
		32		110.0	97.78/12.22	98.7/110.9	8.15	1.1
1.5*h*_0_ (c)	$2\;h_{0}$ (series a)	33	30.5/2.33	139.0	126.41/12.59	121.13/133.7	41.57	35.0
		34		95.0	82.41/12.59	79.23/91.82	−0.3	
		35		125.0	112.41/12.59	108.06/120.65	28.52	
		36		100.0	87.41/12.59	84.03/96.62	4.5	2.1
	$2.5\;h_{0}$ (series b)	37	29.8/2.3	105.0	91.65/13.35	88.46/101.8	23.9	23.9
		38		84.5	71.15/13.35	68.67/82.02	4.12	
		39		105.0	91.65/13.35	88.46/101.8	23.9	
		40		105.0	91.65/13.35	88.46/101.8	23.9	14.0
	$1.5\;h_{0}$ (series c)	41	24.3/2.05	130.0	117.78/12.22	125.25/137.47	34.72	37.4
		42		98.0	85.78/12.22	91.22/103.44	0.69	
		43		135.0	122.78/12.22	130.57/142.79	40.0	
		44		112.75	100.53/12.22	106.9/119.13	16.38	8.5

**Table 3:** Comparison of the average values of the transverse force perceived by U-shaped composite stirrups, depending on the presence of initial inclined cracks and shear spans.

Shear Span during the Formation of Initial Cracks	$\mathrm{Average}\;\mathrm{Shear}\;\mathrm{Force}\;{\bar{Q}}_{f}$ $;\;{\bar{Q}}_{f}^{*}(\mathrm{kN}),\;\mathrm{in}\;\mathrm{Composite}\;\mathrm{Stirrups},\;\mathrm{at}\;\mathrm{Shear}\;\mathrm{Spans}:$		
	$1.5\;h_{0}$	$2.0\;h_{0}$	$2.5\;h_{0}$
1	2	3	4
Elements reinforced with U-stirrups			
$1.5\;h_{0}$	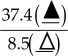	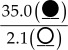	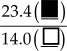
$2.0\;h_{0}$	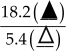	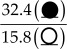	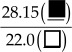
$2.5\;h_{0}$	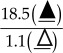	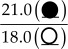	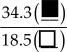
Average for series	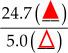	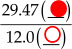	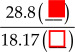

Note. (1) In the numerator of columns 2; 3; 4 shows data for prototypes without initial inclined cracks ${\bar{Q}}_{f}$, and in the denominator, if they are present (${\bar{Q}}_{f}^{*}$). (2) In parentheses for the display on the graphs, the values of the transverse force ${\bar{Q}}_{f}$ and ${\bar{Q}}_{f}^{*}$ are replaced by symbols (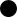 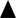 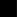); (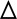 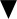 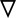) and (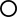 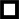), which simultaneously reflect the value of the shear span during testing, the presence or absence of initial cracks, as well as options for external composite reinforcement.

**Table 4:** Comparison of experimental values of transverse forces with theoretical ones for specimens reinforced with external composite reinforcement.

Indicators	$\mathrm{Rate}\;Q_{}^{exp}/Q_{}^{teor}\mathrm{When}\;\mathrm{Calculating}:$			
	SP 164.1325800.2014		Considering the Authors’ Suggestions	
	Elements without Initial Cracks	Elements with Initial Cracks	Elements without Initial Cracks	Elements with Initial Cracks
$\mathrm{Avarage}\;\mathrm{rate}\;\Delta_{cp}=$ $Q_{}^{\exp}/Q_{}^{teor}$	1.450/1.2	1.251/1.042	1.18/1.07	1.099
Standart deviation *S*	0.157/0.131	0.113/0.101	0.122/0.103	0.134
Variation coefficient$\;S/\Delta_{cp}$	0.108/0.109	0.090/0.096	0.104/0.096	0.122

## Data Availability

The study did not report any data.
